# HPV infection and breast cancer risk: insights from a nationwide population study in Taiwan

**DOI:** 10.3389/fonc.2023.1210381

**Published:** 2023-07-14

**Authors:** Chuck Lin, Stella Chin-Shaw Tsai, Jing-Yang Huang, Frank Cheau-Feng Lin

**Affiliations:** ^1^ Department of Biomedical Informatics, Harvard Medical School, Boston, MA, United States; ^2^ Superintendents’ Office, Tungs’ Taichung MetroHarbor Hospital, Taichung, Taiwan; ^3^ Department of Post-Baccalaureate Medicine, College of Medicine, National Chung Hsing University, Taichung, Taiwan; ^4^ Department of Medical Research, Chung Shan Medical University Hospital, Taichung, Taiwan; ^5^ Institute of Medicine, Chung Shan Medical University, Taichung, Taiwan; ^6^ Department of Thoracic Surgery, Chung Shan Medical University Hospital, Taichung, Taiwan; ^7^ School of Medicine, Chung Shan Medical University, Taichung, Taiwan

**Keywords:** breast cancer, cancer risks, human papillomavirus - HPV, population-based study, real-world data (RWD)

## Abstract

**Background:**

The prevalence of cancer, specifically breast cancer, has raised globally. The etiology of breast cancer has been attributed to age, genetic mutations, reproductive history, hormone therapy, lifestyle factors, and viral infections. The human papillomavirus (HPV) has been one of the most widespread sexually transmitted infection in the United States. The role of HPV in breast oncogenesis was hypothesized before, yet the association remained unclear.

**Methods:**

In this study, we employed a nationwide population study using centralized patient data managed by the Ministry of Health and Welfare in Taiwan and the Taiwan Cancer Registry database. The breast cancer incidence rates of the 467,454 HPV patients were compared to twice as many non-HPV patients with matching sex and age. Cumulative breast cancer incidence rates were presented by a Kaplan-Meier curve, and the relative risk of breast cancer for HPV and non-HPV patients were calculated using Cox-regression model.

**Results:**

Our results indicated a crude hazard ratio (HR) and an adjusted hazard ratio (aHR) of 2.336 and 2.271, respectively, when comparing the risk of breast cancer in the HPV and non-HPV group. The risk of breast cancer was comparable or higher than those of head and neck cancer (aHR=1.595) and cervical cancer (aHR=2.225), which both were found to have causal relationships with HPV. The Kaplan-Meier curve further illustrated a higher cumulative risk across 84 months for HPV patients (p<.0001). Besides HPV, age (p<.0001), insurance providers (p<.001), and comorbidities such as abnormal liver function (aHR=1.191, p=.0069) and hyperlipidemia (aHR=1.218, p=.0002) were found to be correlated with higher risks of breast cancer.

**Conclusion:**

A correlation between HPV and breast cancer can be inferred using national health databases. More molecular studies are required to understand the mechanism of the virus-induced oncogenesis of the breast.

## 1 Introduction

Cancer is the leading cause of death and morbidity across the globe. In 2020, approximately 19.3 million new cancer cases were registered worldwide (1), with 122 thousand cases registered in Taiwan, equivalent to 311.34 per 10^6^ person (2). In both populations, the most prevalence cancer for female patients was breast cancer, accounting for 11.7% globally and 12.5% in Taiwan of all reported cancer incidents (1, 2). Globally, around 2.26 million female patients were diagnosed of breast cancer, and approximately 685 thousand cases were fatal in 2020 (1). In Taiwan, about 15.3 thousand females were diagnosed with breast cancer, and around 2.66 thousand cases were fatal (2). Notably, elevated risk factors of breast cancer were reported in patients with lower educational attainment and among racial minorities (3). This finding highlights the need to promote accessible screening services and cancer awareness among individuals in lower socioeconomic statuses.

Factors contributing to the etiology of breast cancer include age (4), genetic mutations (5), reproductive history (6), hormone therapy (7), and lifestyle (8). The role of viral infection in oncogenesis has been examined, and 2.2 million new cancer incidents across the world were related to infection in 2018 (9). Some viruses such as hepatitis B virus and hepatitis C virus can cause chronic inflammation which further cell damages and raise the risks of carcinogenesis (10). Others such as Epstein-Barr virus (EBV) were found to upregulate oncogenes and accelerate cell cycles, leading to rapid cell division and cancer development (11). In addition, EBV was also reported to downregulate the tumor suppressor gene through epigenetic, post-transcriptional, and post-translational modifications (12). Importantly, viruses like human papillomavirus (HPV) can integrate their viral DNA to the host genome, resulting in the dysregulation of cellular growth and the subsequent tumorigenesis (13).

HPV is a sexually transmitted virus characterized as developing genital warts on patients. It is the most prevalent sexually transmitted infection in the world, with a global estimation of 1 in 10 women being HPV carriers at any time (14). The oncogenic property of HPV was prominently shown as the leading cause of cervical cancer, reflected by the HPV vaccination campaigns in multiple countries’ attempts to eliminate cervical cancer in patients across sexual orientations (15, 16). HPV was also shown to cause a proportion of anal (17) and oropharyngeal cancer (18), and served as a known risk factor for head and neck, vulvar, vaginal, and penile cancer, as well as respiratory and laryngeal tumor (19). Other than HPV, human herpesvirus 8 (HHV-8) has been also reported to be associated with breast cancer in which HHV-8 antibodies were found in the blood sera (20). Furthermore, breast cancer might involve multiple viral infections, such as a combination of HSV-1, HPV, HCMV, EBV, and HHV-8, found in breast tissue samples (21).

Currently, the correlation between HPV and breast cancer remained conflicting. Higher HPV viral loads were detected in breast cancer tissues (22), yet a causal relationship was failed to establish between HPV and breast cancer due to inconsistent association scores across studies and the unspecific infective characteristics of HPV (23). Given the currently available evidence, HPV was proposed as a cofactor or mediator in breast cancer etiology (24). Therefore, more large-scale studies are needed to elucidate the association.

## 2 Methods

### 2.1 Data source

Patient data were extracted from a pool of 26 million in the National Health Insurance Research Dataset, 2007-2015, and the Taiwan Cancer Registry, 1979-2015. Both databases were overseen by the Health and Welfare Science Center, Ministry of Health and Welfare, Taiwan. To avoid misclassification of ICD code recorded in the National Health Insurance Research Database and to augment the documentation accuracy of breast cancer, data from the Taiwan Cancer Registry were used to confirm the breast cancer status. Each patient in the datasets possessed an encrypted identification number using their unique Taiwanese National Identification Number. Recorded patient information for each patient included ambulatory care expenditures by visits, inpatient expenditures by admissions, details of ambulatory care orders, details of inpatient orders, registry for beneficiaries, cause of death, and Taiwan Cancer Registry – long/short form.

### 2.2 ICD-9 international classification of diseases, ninth revision

Cases of human papillomavirus (ICD-9-CM 079.4, 078.1, 795.05, 795.09, 795.15, 795.19, 796.75, 796.79) were included and compared with the non-HPV group. Incidences of female breast cancer (ICD-9 174. X), head and neck cancer (ICD-9 140-149. X), and cervical cancer (ICD-9 180. X) were collected. Ischemic heart disease (IHD, ICD-9 411. 413–4), hypertension (ICD-9 401), ischemic stroke (ICD-9: 433–4,436), diabetes mellitus (DM, ICD-9 250), abnormal liver function (ICD-9 571), renal failure (ICD-9 580–589), gastrointestinal (GI) bleeding (ICD-9 578. X), hyperlipidemia (ICD-9 272. X), chronic kidney diseases (ICD-9 585.9), chronic obstructive pulmonary disease (COPD, ICD-9 492-496), peptic ulcer (ICD-9 533), and gout (ICD-9 274. X) were listed as the comorbidities.

### 2.3 Study design and ethical considerations

In the population-based cohort study, the index date was designated as the point of origin, and cancer incidence was measured from the index date until December 31, 2015, with the exclusion of the male population from the cervical cancer statistics. The study protocol underwent review and approval by the institutional review board of Chung Shan University Hospital (IRB CS13168) to ensure adherence to ethical considerations. Patient data from the National Health Insurance Research Dataset were obtained, and de-identification was performed, which waived the need for signed informed consent.

### 2.4 Statistical analysis

Statistical analyses were conducted using SAS 9.4 (SAS Institute Inc., Cary, North Carolina) in this study. HPV-infected cases were matched to non-infected cases according to age, sex, and index date in a 1:2 fashion using structured query language (SQL). Patients with a cancer onset before the index date were excluded from the analysis. Comparisons were made between HPV-infected and non-HPV-infected individuals. Demographic data were analyzed using the chi-square test for categorical data and Student’s t-test for numerical data. We used the multivariate Cox regression to adjust the potential confounding effect of age, urbanization, insured type, co-morbidities (including ischemic heart disease, hypertension, ischemic stroke, diabetes mellitus, abnormal liver function, renal failure, gastrointestinal bleeding, hyperlipidemia, chronic kidney diseases, chronic obstructive pulmonary disease, peptic ulcer, and gout) to estimate the hazard ratio of breast cancer in patients with HPV infection compared with the non-HPV individuals. We used the Schoenfeld residuals to test the proportional hazard of breast cancer, and the assumption of proportional hazard was not violated. Additionally, Kaplan-Meier curves were utilized to generate cumulative incidence rates of cancer, which were then tested using the log-rank test.

## 3 Results

**Figure 1:**
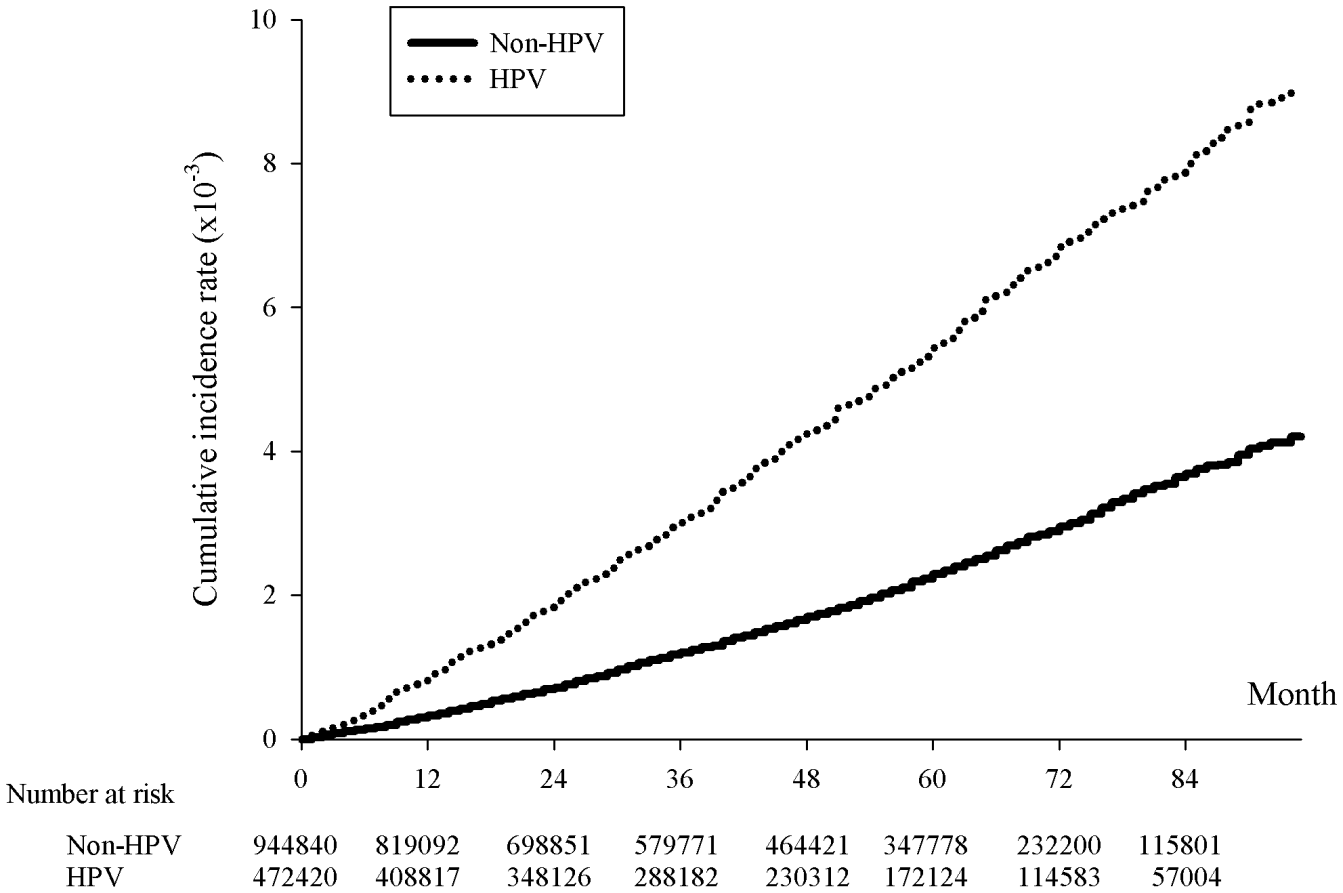
Kaplan-Meier curve of the cumulative incidence rates of breast cancer with and without HPV infection. The x-axis represents month since the index date, supplemented with the number of HPV cases and twice the non-HPV cases collected within each span of twelve months, p<.0001. The y-axis indicated the incidence rates in 10^-3^.

About 26 million patients were registered in the National Health Insurance Research Datasets in Taiwan between 2007 to 2015. Among them, 1,103,771 patients were once diagnosed with HPV infection. Patients who had prior history of HPV infection before 2008 were excluded from the study, so were those who developed cancer before the HPV infection. This resulted in a pool of 939,874 HPV patients included in this study [**Figure 1** (25)]. As the control, twice as many non-HPV patients with matching age, sex, and index date as the HPV patients (p=1.0000) were selected from the rest of the 25,462,267 non-HPV patients in the National Health Insurance Research Dataset using Structured Query Language (SQL) (**Table 1**). Nevertheless, several demographic characteristics differed between the HPV positive and negative groups, such as the geological distribution of patients (p<0.0001), the regional levels of urbanization (p<0.0001), the insurance providers (p<0.0001), and several co-morbidities such as ischemic heart disease (p<0.0001), hypertension (p<0.0001), diabetes mellitus (p<0.0001), renal failure (p<0.0001), GI bleeding (p=0.0006), hyperlipidemia (p<0.0001), chronic kidney diseases (p<0.0001), COPD (p<0.0001), peptic ulcer (p<0.0001), and gout (p<0.0001). Subsequently, the breast cancer patients were identified from this pool of HPV positive and negative patients and cross-examined with the Taiwan Cancer Registry to validate the breast cancer status of each selected patient.

**Table 1:** Baseline characteristics of the HPV and non-HPV populations.

	Non-HPV	HPV	p
**Sex**			1.0000
Male	944,840 (50.26%)	472,420 (50.26%)	
Female	934,908 (49.74%)	467,454 (49.74%)	
**Age**			1.0000
<20	513,000 (27.29%)	256,500 (27.29%)	
20-40	669,372 (35.61%)	334,686 (35.61%)	
40-60	469,150 (24.96%)	234,575 (24.96%)	
60-80	191,510 (10.19%)	95,755 (10.19%)	
>=80	36,716 (1.95%)	18,358 (1.95%)	
**Urbanization**			<.0001
Urban 1	542,433 (28.86%)	319,694 (34.01%)	
2	587,017 (31.23%)	290,511 (30.91%)	
3	340,505 (18.11%)	162,859 (17.33%)	
4	246,844 (13.13%)	109,824 (11.68%)	
5	36,687 (1.95%)	13,197 (1.40%)	
6	70,850 (3.77%)	23,620 (2.51%)	
Rural 7	55,412 (2.95%)	20,169 (2.15%)	
**Geographic regions**			<.0001
Taipei metropolitan	671,431 (35.72%)	395,713 (42.10%)	
Northern	279,894 (14.89%)	124,964 (13.30%)	
Central	347,664 (18.50%)	180,479 (19.20%)	
Southern	261,434 (13.91%)	107,447 (11.43%)	
Kaohsiung and Pingtung	276,130 (14.69%)	115,919 (12.33%)	
Eastern	43,195 (2.30%)	15,352 (1.63%)	
**Insurance Provider**			<.0001
Civil Servants’ Insurance	111,753 (5.95%)	79,002 (8.41%)	
Labor Union	1,183,084 (62.94%)	618,860 (65.84%)	
Farmers’, Fisherman’s Association and the water conservancy	237,256 (12.62%)	94,224 (10.03%)	
Low-income household’s insurance	20,772 (1.11%)	7,928 (0.84%)	
Township office	294,431 (15.66%)	122,182 (13.00%)	
Others	32,452 (1.73%)	17,678 (1.88%)	
Co-morbidity			
Ischemic heart disease	63,872 (3.40%)	39,334 (4.19%)	<.0001
Hypertension	205,015 (10.91%)	112,709 (11.99%)	<.0001
Stroke	40,087 (2.13%)	20,246 (2.15%)	0.2386
Diabetes mellitus	96,584 (5.14%)	46,782 (4.98%)	<.0001
Abnormal liver function	91,126 (4.85%)	45,661 (4.86%)	0.7009
Renal failure	15,200 (0.81%)	8,191 (0.87%)	<.0001
GI bleeding	10,826 (0.58%)	5,723 (0.61%)	0.0006
Hyperlipidemia	133,790 (7.12%)	83,114 (8.84%)	<.0001
Chronic kidney diseases	25,740 (1.37%)	14,747 (1.57%)	<.0001
COPD	32,326 (1.72%)	18,607 (1.98%)	<.0001
Peptic ulcer	122,250 (6.50%)	74,302 (7.91%)	<.0001
Gout	49,184 (2.62%)	28,685 (3.05%)	<.0001

The incidence rate of breast cancer on women was 109.67 per 100000 person-years for HPV patients, comparing to 46.97 per 100000 person-years for non-HPV patients, giving a crude hazard-ratio (HR) of 2.336 and an adjusted HR (aHR) of 2.271 (**Table 2**). Not only did this indicate that the prevalence rate of breast cancer was higher for HPV than non-HPV patients, but the risk of breast cancer was also equivalent or higher than the risk of head and neck cancer and cervical cancer in HPV patients, reflected by an approximately 42% and 2% higher aHR of breast cancer than that of head and neck cancer and cervical cancer, respectively, between HPV and non-HPV patients (**Table 2**). The cumulative breast cancer incidence rate over the span of 84 months was significantly higher in HPV positive than negative group as shown in the Kaplan-Meier curve (p<0.001) (**Figure 1**).

**Table 2:** Risks of cancers.

	Non-HPV			HPV				
	Pm	Event	Incidence rate†	Pm	Event	Incidence rate†	Crude HR	Adjusted HR
Female breast cancer	45,020,697	1,762	46.97 (44.82-49.21)	22,388,511	2,046	109.67 (105.01-114.52)	2.336(2.192-2.490)	2.271(2.129-2.421)
Head and neck cancer	89,136,676	1,064	14.32 (13.49-15.21)	44,357,317	808	21.86 (20.40-23.42)	1.527(1.393-1.673)	1.595(1.453-1.749)
Cervical cancer	45,020,697	712	18.98 (17.63-20.42)	22,388,511	777	41.65 (38.82-44.68)	2.195(1.983-2.430)	2.225(2.008-2.464)

Pm, person-months.

† Crude incidence rate, per 100000 person-years.

Besides HPV infection with a 2.271 aHR (p<0.0001), other demographic factors might also correlate with the breast cancer incidence rate. The biggest factor was age, where the highest aHR was 4.938 in the group aged 40 to 60 (p<0.0001), followed by 4.058 in the group aged 60 to 80 (p<0.0001), then 1.937 in group aged over and equal to 80 (p<0.0001), and finally 0.011 in group aged less and equal to 20 (p<0.0001), with the age group 20 to 40 served as the control (**Table 3**). In addition, the breast cancer incidence rates varied among patients covered under different insurance providers. Patients covered by the civil servants’ insurance had a higher aHR of 1.234 (p=0.0002), whereas those covered by the insurance of Farmers’, Fisherman’s Association and the Water Conservancy had a lower aHR of 0.796 (p=0.0009) (**Table 3**). The incidence rates also differed among breast-cancer patients with co-morbidities. Significantly, the incidence rates of breast cancer were higher in patients experiencing abnormal liver function (aHR=1.191, p=0.0069) and hyperlipidemia (aHR=1.218, p=0.0002).

**Table 3:** Adjusted hazard ratios for breast cancers.

	aHR	95% CI	p
**HPV**	2.271	2.129-2.421	<.0001*
Sex			
Male			
Female			
Age			
<20	0.011	0.005-0.027	<.0001*
20-40	Reference		
40-60	4.938	4.518-5.397	<.0001*
60-80	4.058	3.594-4.581	<.0001*
>=80	1.937	1.429-2.624	<.0001*
Urbanization			
1	Reference		
2	0.983	0.903-1.071	0.6967
3	0.887	0.794-0.990	0.0324
4	0.927	0.816-1.053	0.2442
5	0.714	0.510-1.000	0.0503
6	0.841	0.659-1.073	0.1633
7	0.890	0.687-1.152	0.3755
Geographic regions			
Taipei metropolitan	Reference		
Northern	0.978	0.874-1.095	0.704
Central	0.938	0.847-1.038	0.2156
Southern	0.884	0.785-0.996	0.0428
Kaohsiung and Pingtung	0.934	0.840-1.038	0.2064
Eastern	0.929	0.721-1.197	0.5685
Insurance Provider			
Civil servants’ insurance	1.234	1.104-1.379	0.0002*
Labor Union	Reference		
Farmers’, Fisherman’s Association and Water Conservancy	0.796	0.695-0.911	0.0009*
Low-income household’s insurance	0.853	0.565-1.287	0.4492
Township office	1.009	0.919-1.108	0.8532
Others	1.054	0.841-1.321	0.6485
Co-morbidity			
Ischemic heart disease	0.968	0.836-1.121	0.6607
Hypertension	1.095	0.994-1.207	0.065
Stroke	1.114	0.922-1.345	0.2635
Diabetes mellitus	1.084	0.957-1.228	0.206
Abnormal liver function	1.191	1.049-1.351	0.0069*
Renal failure	1.123	0.694-1.818	0.6357
GI bleeding	0.829	0.506-1.359	0.4574
Hyperlipidemia	1.218	1.097-1.353	0.0002*
Chronic kidney diseases	1.027	0.711-1.484	0.8876
COPD	1.107	0.882-1.390	0.381
Peptic ulcer	1.071	0.966-1.188	0.1939
Gout	1.074	0.848-1.360	0.5545

Cox regression.

*p < 0.01.

## 4 Discussion

This study presented a positive association between HPV infection and the risk of breast cancer using national population data from the Taiwanese single-payer healthcare registry over 84 months. The scale of this study provided an advantage to counter the inconsistency of the past results attempting to associate HPV infection and breast cancer. The 1:2 HPV positive to negative study group design also strengthened the statistical power of the association. Additionally, our study included multiple demographic factors that might serve to offer further insights into the correlation.

The most common transmission route of HPV is through sexual activities. Nevertheless, other transmission mechanisms have been proposed that related to the contact of skin and mucus, including horizontal transfer through non-sexual contact of fomites, fingers, skin, and mouths, self-inoculation presented by female virgins and child with absence of sexual abuse history, and vertical transmission during childbirth (26). How HPV travelled to and resided in breast tissues remained unclear. One theory suggested that HPV can be transmitted from the primary tumor such as cervical neoplasm to the mammary glands through plasma circulation (24). Another proposed that the viral particle can enter the milk ducts and populated in the mammary glands by the means of direct or hand-mediated sexual contact.

On the cellular level, HPV infected the cellular membrane and inserted the L2 capsid proteins into endosome facilitated by a transmembrane protease. The endosome displaying L2 protein protrusion was then trafficked to the Golgi apparatus assisted by cytosolic host factors (27). HPV DNA was then sent to microtubule-organizing center and, finally, it was shipped to chromosome *via* kinesins and spindle fibers during metaphase and anaphase (28).

Molecular studies have also indicated the association between HPV and breast cancer by attempting to provide a plausible transmission mechanism. The comparison between HPV positive and negative breast cancer tissues revealed a downregulation of p53 and an upregulation of BCL2, a hallmark of uninhibited cellular checkpoints (29). The phosphorylation of Erk1/2 and ß-catenin pathway might also be enhanced in breast cancer tissue when HPV L6/L7 cooperated with LMP1 oncoproteins, leading to cell proliferation (30). The proinflammatory cytokine IL-6, which was reported to progress oncogenesis, exhibited increased expression in breast cancer patients with HPV (31). Lastly, the blood-transmission theory of HPV was further supported by the finding of HPV DNA in extracellular vesicles extracted from the serum of breast cancer patients (32).

Other factors obtained from the patient data might confound the association of HPV and breast cancer. Our study reported that age, insurance provider, and co-morbidities such as abnormal liver function and hyperlipidemia influenced the breast cancer incidence rate. Among these confounding factors, our study indicated that the highest risk of breast cancer in the female population was between the ages 40 to 60, compared to all cancer whose risk retained positive correlation across age groups. We postulate that since most menopause occurred at the age of 40 to 60 where hormone homeostasis readjusted and HPV infection rate could also positively associate with menopausal status and negatively with hormone replacement therapy (33), breast cancer incidence rate was likely to be affected by HPV infection due to the alteration of hormone levels. On one hand, the decreased level of estrogen during menopause might result in virginal microbiomes being more favorable for HPV infection (34). On the other hand, molecular studies indicated the potential association of estrogen and apolipoprotein B messenger RNA-editing, enzyme-catalytic, polypeptide-like 3 (APOBEC3) family of cytidine deaminases, which served not only to induce antiviral immune response during the HPV infection, but also could mutate host DNA and initiate breast carcinogenesis (35). Estrogen and the lack of p53 were reported to potentially upregulate APOBEC enzymes synergistically in the breast cancer cells containing estrogen receptors (35, 36).

Another confounding factor, the abnormal liver function could be potentially elucidated by the presence of HPV DNA circulating to liver. HPV was found to act cooperatively with hepatitis B virus (HBV) to develop hepatocellular carcinoma, and/or other viral infections might be facilitated by HPV to cause abnormal liver function (37).

The current theory proposed that HPV may be a cofactor or mediator of breast cancer rather than a causative agent, partially due to conflicting results of the presence of HPV in breast cancer tissues (24). Moreover, demographic and other individual factors may influence the possibility of oncogenesis after HPV infection. Our result was consistent with this theory and multiple demographic factors were examined in **Table 3**. The association of HPV infection status and breast cancer was further supported by increased breast cancer rate found in patients infected by high-risk HPV, including HPV 16, 18, and 33, in a large-scale meta-analysis study (38). Additionally, the association was verified by four different PCR approaches for HPV detection (39). In contrast, studies in different populations across the globe did not yield statistically significant results unanimously to indicate the contribution of HPV on breast cancer development (40, 41). However, the lack of HPV prevalence in breast cancer tissue could potentially be explained by the disappearance of HPV strains in the later stage of cancer (24).

A main obstacle was to understand the route of HPV transmission to breast tissues. Unlike cervical cancer, where HPV infected the epithelial cells of the cervix *via* cervical lesion or the mucous membrane, HPV had less direct routes to infect breast tissues. Although two potential mechanisms where proposed above (24), more anatomic and molecular studies are required to understand the direct and indirect etiologies of HPV on breast cancer.

In summary, our results suggested a significant higher risk of breast cancer in female patients with HPV than those without. The risk of breast cancer in HPV positive patients was reported to be twice as large as HPV negative patients. The accumulated breast cancer incidence rates between HPV positive and negative patients were shown to be significantly different in Kaplan-Meier curve. Despite not being able to conclude a causal relationship, it could be assumed the role of HPV as a contributor of the breast carcinogenesis.

Interestingly, our analysis on patient demographics speculated a potential role of estrogen between HPV infection and breast cancer, which could only be explained by executing further investigations. The circulation of HPV in sera might also implicate other organs such as the liver, but it is beyond the scope of this study.

The hypothesis that breast cancer was associated with HPV was first proposed about 30 years ago (24). However, the link has not been strong enough due to mixed results of HPV prevalence rates in breast cancer patients across studies. Our study utilized large-scale datasets to provide a more robust statistical significance between the incidence rates of breast cancer and HPV status. 26 million people were registered in the Taiwanese Health Registry over the year 2007 to 2015. The breast cancer statuses of the patients were identified in the registry and verified by another database, Taiwanese National Cancer Registry.

It is recognized that a limitation of the study was that the subtype and localization of the HPV was unknown. Investigating the HPV subtype with respect to breast cancer incidence rate may provide clues about the oncogenic properties of high-risk HPV subtype, while data on HPV localization may suggest its possible infectious mechanism in the development of breast cancer. Additionally, it is worth noting that the population-based data used in this study were not collected solely for the purpose of this research. As a result, there is a potential for some ICD outcomes to have been misclassified. Moreover, confounding variables previously reported to be associated with the risk of breast cancer, such as reproductive history, breast feeding, family history of breast cancer, lifestyle, and environmental factors, were not fully accounted for in this study due to the inherent nature of the data collection, which may cause bias in the results. Finally, the results of the study should be interpreted with caution, as the risk factors for breast cancer may differ in different populations.

Nevertheless, a causal relationship between HPV and breast cancer remains unclear. More hypotheses proposed from molecular studies are required to understand the route of HPV transmission to breast tissues as well as the mechanism of viral transmission in breast cells in relation to oncogenesis. Moreover, the HPV subtypes of the breast cancer patients could be examined in the future to provide insights into the mixed HPV prevalence rates in the previous studies of breast cancer tissues. Furthermore, the societal impacts of cancer treatments are becoming more prominent each year. Not only the national treatment cost for cancer was estimated to be 246 billion in the United States by 2030 (42), but also cancer patients pay four times more than patients without cancer on average (43), in addition to the higher risks of psychiatric disorders and mental distress on both cancer patients and their nuclear family members (44). Thus, our study included HPV status, age groups, co-morbidities, and other demographic variables to provide evidence supporting a more stringent approach to breast cancer screening. More research could be done to investigate these factors which could be valuable in drafting novel health initiatives to combat breast cancer.

## Data availability statement

The original contributions presented in the study are included in the article/supplementary material. Further inquiries can be directed to the corresponding author.

## Author contributions

Conception or design of the work: J-YH and FC-FL. Data collection: J-YH, CL, SC-ST, FC-FL. Data analysis and interpretation: J-YH, FC-FL. Drafting the article: CL. Critical revision of the article: SC-ST, FC-FL. Approval of the manuscript: J-YH, CL, SC-ST, FC-FL. All authors contributed to the article and approved the submitted version.
